# Analysis of Nutrient Composition, Rumen Degradation Characteristics, and Feeding Value of Chinese Rye Grass, Barley Grass, and Naked Oat Straw

**DOI:** 10.3390/ani11092486

**Published:** 2021-08-24

**Authors:** Yulin Ma, Muhammad Zahoor Khan, Yanfang Liu, Jianxin Xiao, Xu Chen, Shoukun Ji, Zhijun Cao, Shengli Li

**Affiliations:** 1 State Key Laboratory of Animal Nutrition, Beijing Engineering Technology Research Center of Raw Milk Quality and Safety Control, College of Animal Science and Technology, China Agricultural University, Beijing 100193, China; ma18810318038@163.com (Y.M.); zahoorkhattak91@yahoo.com (M.Z.K.); 13611235024@163.com (Y.L.); dairyxiao@gmail.com (J.X.); sy20193040671@cau.edu.cn (X.C.); caozhijun@cau.edu.cn (Z.C.); 2 College of Animal Science and Technology, Hebei Agricultural University, Baoding 071001, China; jishoukun@163.com

**Keywords:** Chinese rye grass, barley grass, naked oat straw, rumen degradation rate, feed value

## Abstract

**Simple Summary:**

Limited studies have been reported so far on the rumen degradability characteristic of low-quality forage. In the current study, three low-quality forages (Chinese rye grass, barley grass, and naked oat straw) were selected. Various nutritional parameters such as rumen degradation characteristics, chemical composition and feeding value of three roughages were studied in detail. Among the three roughages, the Chinese rye grass (*Leymus chinensis)* was superior in terms of rumen degradability characteristics, chemical composition and feeding value.

**Abstract:**

The current study was designed to investigate the chemical composition, rumen degradation characteristics, and feeding value of three roughages commonly used in Asia as ruminant feed, including Chinese rye grass (CRG), barley grass (BG), and naked oat straw (NO). Four Holstein Friesian cows equipped with permanent rumen fistulas were chosen for experimental trials in the current study. The nylon bag method was carried out to measure the crude protein (CP), acid detergent fiber (ADF), ruminal degradability of dry matter (DM), and neutral detergent fiber (NDF). Our analysis revealed that the contents of CP in the CRG (9.0%) and BG (8.9%) were higher than in the NO (5.94%). The contents of NDF in the CRG (64.97%) and NO (63.83%) were lower than in the BG (67.33%), and the content of ADF in the CRG (37.03%) was lower than in the BG (37.93%) and NO (38.28%). The ED values of DM in the NO and CRG were significantly higher (*p* < 0.001) than in the BG. The effective degradability (ED) values of NDF were the highest in the CRG and lowest in the NO (*p* < 0.001). In addition, the ED values of ADF were the highest in the CRG and lowest in the BG (*p* < 0.001). The ED value of CP in the CRG was significantly higher than that in the BG and NO (*p* < 0.001). The estimated total digestible nutrients (TDN) (54.56%) and DM degradation rate (DDM) (60.06%) of the CRG were higher than those of BG and NO. In addition, the expected DM intake (DMI), estimated relative feed value (RFV), and estimated relative feed quality (RFQ) of the BG were lower than those of the CRG and NO. Altogether, based on our findings, we concluded that the nutritional quality, feeding value and effective rumen degradation rate of CRG were better than of BG and NO.

## 1. Introduction

High-quality forage is essential for the production performance and health of ruminants [1]. At present, there is a serious shortage of high-quality forages in the ruminant industry in China [2]. Chinese rye grass *(Leymus chinensis)* (CRG) is a perennial grass with the characteristics of good palatability and strong resistance to trampling. The CRG is widely distributed in the eastern Inner Mongolian plateau and Songnen plain in China [3]. The feed value of CRG product has also been reported in ruminants’ nutrition, while the harvesting time of CRG has shown a significant effect on dry matter digestibility and feed intake in sheep. Furthermore, it has been documented that harvesting of CRG in spring and summer showed greater dry matter digestibility and feed intake in sheep than in the autumn and winter [4,5,6]. The combination of *Urtica cannabina* L. and CRG at the rate of 50:50 had shown greater in vitro digestibility of neutral detergent fiber (NDF) than at the ratio of 0:100, 30:70, 70:30, and 100:0 [7]. Moreover, a positive effect of CRG on the growth performance for ewes has been reported in winter in a pastoral-farming area of northeast China [8]. Consequently, a study reported that 50% dry matter (DM) digestibility of CRG hay [9], while 38.55% to 54.44% digestibility of CRG organic matter has been documented by Sun et al. [4]. The palatability and high yield characteristics of CRG make it ideal for both grazing and hay production. Thus, CRG is considered an important source of forage for grassland grazing or house-raising livestock in northern China. The nutritional value evaluation of CRG is important for grassland management, grazing, and nutrition management of house-raising livestock. However, there is limited information on the evaluation of feed nutritional value. Therefore, in order to fully develop ruminant feed resources, it is very necessary to understand the nutritional characteristics and digestibility of CRG.

Barley grass (*Hordeum violaceum Boiss*) mainly grows in high altitude areas and is planted in two distinct seasons. A study reported that the content of CP in Barley grass (BG) was 5.8%, which is the protein level of low-quality roughage [10]. Fay et al. [11] demonstrated that NDF and ADF contents of BG were 58.54% and 35.39%, respectively [11]. Consistently, a study documented that the potential degradation and total degradation of barley grass (BG) were 168 g/kg DM and 412 g/kg DM, respectively [12]. Consistently, 49.9%, 47.0%, and 52.3% of digestibility has been documented for DM, organic matter (OM), and ADF, respectively in BG [13]. At present, there is a shortage of roughage resources for ruminants [14]. Therefore, evaluating the nutritional value of BG can provide basic data for the better utilization of roughage resources.

Naked oat (*Avena nuda* L.) is an annual grass species native to China that is different from hull oat (*Avena sativa* L.) in terms of morphology and nutritional value. According to the survey by Food and Agriculture Organization in 2017, the annual production of naked oat straw (NO) was approximately 1.4 million tons in China [15]. A study reported that the content of CP in NO was 16.5% [10], which means that NO is a potential high-protein roughage. Li et al. [15] documented that the content of CP and crude fiber in NO were 3.3% and 45.2%, respectively [16]. This may be due to the different CP content in NO in different regions. Zheng et al. [15] showed that the in vitro DM digestibility (IVDMD), in vitro NDF digestibility (IVNDFD), and in vitro ADF digestibility (IVADFD) of NO were 395.1 g/kg, 302.5 g/kg, and 288.0 g/kg, respectively [17]. The digestibility data of these nutrients suggest that NO could become a potential source of roughage.

Being a high potential value as forage, further studies on the nutrition value of CRG, BG, and NO would be helpful to understand their utilization in ruminants as a forage resource. A study has documented that the degradation characteristics of the three forages in the rumen are important indicators for evaluating the nutritional value [18]. Nylon bag method is the primary technique used to evaluate the degradation of ruminant feed [19]. Very limited data is available on the rumen degradability characteristic of low-quality forages, especially of CRG, BG, and NO. Therefore, we designed the current study with the aim to investigate the chemical composition, rumen degradation characteristics, and feeding value of CRG, BG, and NO.

## 2. Materials and Methods

### 2.1. Raw Materials 

The details of three types of forages (CRG, BG, and NO) have been given in Table 1. The CRG, BG, and NO samples were oven-dried at 65 °C for 48 h, and portions of the air-dried samples were ground through a 1.0 mm aperture sieve to detect chemical components. To determine rumen degradation characteristics, the other parts were milled through a 2.5 mm sieve (Aizela electric appliance co. LTD., Zhejiang, China). 

### 2.2. Animals and Experimental Design

Four Holstein Friesian cows (body weight: 600 ± 11.8 kg; the average dry matter intake: 25 ± 2 kg/day·cow; lactation number: 2; day of lactation was 152 ± 14 and milk yield 37 ± 4 kg/day) equipped with permanent rumen fistulas were used to measure the ruminal degradability of nutrient of three forages by nylon bag method. The cow’s basic diet and nutrient levels are provided in Table 2.

### 2.3. Chemical Analysis

Three different forage raw materials were selected, and each of forage was sampled in triplicate. Furthermore, the nine samples were oven-dried at 65 °C for 48 h to a constant weight to analyze the DM contents, and then ground in a hammer mill to pass a 1 mm sieve for chemical analysis. The crude protein (CP), crude ash (Ash), and starch content of the samples were analyzed using the procedures recommended by AOAC (1984) [20]. The content of neutral detergent fiber (NDF) and acid detergent fiber (ADF) in three forage samples were analyzed according to the method adopted by Van Soest et al. [21]. The content of NDF and ADF was measured by using the ANKOM 2000i automatic fiber analyzer (Beijing Anke Borui Technology Co. Ltd., Beijing, China). 

### 2.4. In Situ Nutrient Degradability

The nylon bag method [22] was performed to assess the rumen degradation characteristics of DM, CP, NDF, and ADF of three kind of roughages. The bag size was 8 × 12 cm with a pore size of 50 um (Ruitong Biotech Co., Ltd., Xinjiang, China). The CRG, BG, and NO samples were milled through a 2.5 mm sieve. Five grams of each sample was weighed and sealed in each nylon bag. Consequently, a polyethylene plastic tube with a length of approximately 50 cm was prepared—two gaps of approximately 1 cm cut at a distance of 3 cm from the port and a small hole drilled at the other end—and the nylon bag containing the samples was fixed on the soft rubber stopper with rubber bands. The other end of the tube was tied with a nylon rope, approximately 20 cm in length, through a small hole. The polyethylene plastic tube with the bag fixed was fed into the rumen one hour before the morning feeding. Each forage sample was put into the rumen of 4 cows (each cow had two parallel bags of each forage at a given time point). All the samples were incubated in the rumen for 4 h, 8 h, 12 h, 24 h, 30 h, 36 h, 48 h, and 72 h. There were 48 bags in the rumen of each cow for all time points. The two bags of each forage sample from the rumen were taken out at each time point; that is, 8 bags were taken out from the rumen of each cow at each time point, and were continued till 72 h.

After removal from the rumen, the nylon bags were rinsed quickly under the water to prevent fermentation. Consequently, the nylon bags were washed with tap water till the water ran clear. Furthermore, the nylon bags were oven-dried at 65 °C for 48 h to constant weight and regain moisture for 24 h and then ground using a ball mill to pass through a 1 mm screen. Then, they were thoroughly mixed, and the DM, CP, NDF, and ADF of samples were analyzed using the method previously described. The degradability value at time 0 was obtained by rinsing four bags per sample. The real-time degradation rate is calculated by the difference subtraction method, and the calculation formula is: the real-time degradation rate of a certain nutrient component of the feed sample (%) = 100 × (the nutrient content before degradation − the nutrient content after degradation)/the nutrient content before degradation.

### 2.5. Calculation of In Situ Nutrient Degradability

The degradation data were fitted to the following exponential equation [23]:

P = a + b (1 − e^−ct^) (1)

P is the nutrient disappearance rate in the rumen at a time “t”; “a” is a rapidly degradable fraction; “b” is the potentially degradable fraction; “c” is the constant rate of degradation of “b”(%/h); and “e” is the base of natural logarithms. “a + b” is the total degradable fraction. The NLIN program in SAS (9.2) was used to calculate the values of a, b, and c. Each cow is regarded as a duplicate; there were two parallel bags in each cow.

Then, the effective degradability (ED) of DM, CP, NDF, and ADF of forage samples were calculated by applying the following equation [23]:

ED(%) = a + b × c/(k + c)(2)

a, b and c are the same parameters represented in Equation (1), and “k” is the rumen outflow rate of the nutrient component (%/h). In this study, the value of k was 3.1%/h [24]. The NLIN program in SAS (version 8.2; SAS Institute, Inc., Cary, NC, USA) was used to calculate the values of a, b, and c.

### 2.6. Feeding Value Evaluation

The quality and expected feed intake of roughage—RFV% = DMI × DDM/1.29—was measured according to the relative value of roughage [25]. The unit for DMI is % of BW (bodyweight) and was only calculated according to the weight of each animal. Relative feed value (RFV) was proposed by the American Pasture and Grassland Council [25]. The other indicators examined in our study were: relative forage quality (RFQ): RFQ% = DMI × TDN/1.23 [26]; total digestible nutrients (TDN): TDN% = 82.38 − (0.7515 × ADF) [25]; dry matter intake (DMI): DMI% = 120/NDF [25]; digestible dry matter (DDM): DDM% = 88.9 − 0.779 × ADF [25].

### 2.7. Statistical Analysis

Data on chemical composition and feed value were analyzed by mean value. Real-time degradability was analyzed using repeated measure data of a MIXED procedure of SAS (9.2) with the model (1).

Yijk =μ + Fi + Tj + (FxT)ij+ Aik + εijk(3)

where μ is the overall mean, Fi is the fixed effect of forage, Tj is the fixed effect of incubation time, and (FxT)ij is the fixed interaction effect between forage and incubation time. Ak is the random effect of animal (k = 4), and εijk is the error.

For all statistical analyses, significance was declared at *p* < 0.05. Differences among the three forages were evaluated using a multiple comparison test following the Tukey–Kramer method.

## 3. Results

### 3.1. Chemical Composition

The chemical compositions of the three forages are shown in Table 3. The DM content of the three forages is between 90.66–90.95%. Additionally, CP content of the CRG and BG was higher than of the NO, which was 9.0% and 8.9%, respectively. The NDF content of the CRG (64.97%) and NO (63.83%) were lower than of the BG (67.33%). The ADF content of the CRG (37.03%) was lower than of the BG (37.93%) and NO (38.28%). Consistently, the ash content of the BG (8.53%) was higher than of the CRG (8.04%) and NO (7.99%). In addition, the starch content was the highest in the CRG (5.64%) and lowest in the BG (2.05%).

### 3.2. Ruminal DM Degradation

The DM degradations of the BG and NO were significantly (*p* < 0.001) higher than of the CRG at 4 h, 8 h, 12 h, 24 h, and 36 h in rumen (Table 4). All the forages degraded faster before 24 h and then leveled off. Compared to the CRG and NO, the DM degradation of 30 (*p* < 0.001) and 48 h (*p* = 0.002) in the BG was highest. The rapidly degradable fraction (a) of the BG was significantly higher (*p* < 0.001) than of the CRG and NO, and no difference was found between the CRG and NO (*p* > 0.05). The constant rate (c) (*p* = 0.005) of the potentially degradable fraction and the potentially degradable fraction (b) (*p* < 0.001) of DM in the BG was significantly lower than in the CRG and NO. The effective DM degradation (ED) of the BG forage was lowest (*p* < 0.001) compared with the CRG and NO, and there was no significant difference (*p* > 0.05) between the CRG and NO.

### 3.3. Ruminal NDF Degradation

The NDF degradation at 8 h, 12 h, and 24 h in the CRG was the highest (*p* < 0.001) compared to BG and NO (Table 5). The NDF degradation of 36 h (*p* = 0.031) and 48 h (*p* < 0.001) in the CRG and BG was significantly higher than in the NO, and no difference was found between the CRG and BG (*p* > 0.05). The NDF degradation of 72 h in the BG was highest (*p* < 0.001) compared to the CRG and NO. Compared with the CRG and NO, the potentially degradable fraction (b) of NDF in the BG was highest (*p* < 0.001). The constant rate (c) of potentially degradable fraction (b) of NDF in the CRG and NO were significantly higher (*p* = 0.01) than in the BG, and no difference was found between the CRG and NO. The effective NDF degradation (ED) was the highest in the CRG and lowest in the NO (*p* < 0.001)

### 3.4. Ruminal ADF Degradation

The ADF degradation at 4 h, 12 h, and 24 h in the CRG was significantly higher (*p* < 0.05) than in the BG and NO (Table 6), and there was no significant difference between BG and NO (*p* > 0.05). The ADF degradation of 30 h, 36 h, 48 h, and 72 h in the CRG and BG was significantly higher (*p* < 0.05) than in the NO, and no difference was found between the CRG and BG (*p* > 0.05). The ADF potentially degradable fraction (b) was the highest in the BG and lowest in the NO (*p* < 0.001). The effective ADF degradation (ED) was the highest in the CRG and lowest in the BG (*p* < 0.001).

### 3.5. Ruminal CP Degradation

The CP degradation at 4 h, 30 h, and 72 h in the CRG was the highest (*p* < 0.05) compared with the NO and BG (Table 7). Compared with the CRG and NO, the rapidly degradable fraction (a) of CP in the BG was the lowest (*p* < 0.001), and no difference was found between the CRG and NO (*p* > 0.05). The potentially degradable fraction (b) of CP in the CRG and BG was significantly higher (*p* = 0.002) than in the NO, and no difference was found between the CRG and BG (*p* > 0.05). The effective degradation (ED) of CP in the CRG was significantly higher (*p* < 0.001) than in the BG and NO, and no difference was found between the BG and NO (*p* > 0.05).

### 3.6. Feed Value Evaluation

The TDN (54.56%) and DDM (60.06%) of the CRG were higher than of the BG (TDN: 53.87%, DDM: 59.35%) and NO (TDN: 53.87, DDM: 59.35%). The DMI (1.78%), RFV (82.02%), and RFQ (78.09%) of the BG were lower than of the CRG (DMI: 1.85%, RFV: 85.99%, RFQ: 81.92%) and NO (DMI: 1.88%, RFV: 86.11%, RFQ: 81.95%). The estimated feeding value of three forages (CRG, BG, and NO) has been summarized in Table 8.

## 4. Discussion

Chinese rye grass (CRG) is an ideal pasture for grazing and hay production due to its good palatability, strong resistance to trampling, and high protein content [27]. The CP contents of the CRG vary greatly in different studies, approximately ranging from 6.36–9.26 [27,28]. The CP contents of the CRG and BG forages were higher than of the NO in our findings, which were 9.0% and 8.9%, respectively. The harvest time of forage grass is one of the important factors affecting CP contents. A study reported that the content of CP was higher and the content of fiber was lower upon initial mowing from 15 May to 15 June (heading), while the opposite results were obtained when the initial mowing was delayed (1 July to 30 September, which belong to bloom to post fruit vegetative period) [29]. The CRG was mowing in the initial bloom stage in this study; and the result related to the content of CP was consistent with the findings documented in the previous study. Meanwhile, a study reported that the CP content of the BG was 5.8% [30], which is lower than the 8.9% documented in the current study. In contrast, a study reported that the content of CP was 14.3% in the BG [31], which might be due to the difference in the harvest season and the maturity period. Consequently, the content of CP in the BG gradually decreased from the heading stage to the milk stage [32]. There was limited information on the content of CP in NO.

The CP content in forage is the most limiting nutrient parameter [33], and some studies have reported that the rumen microbial activity was inhibited and the forage intake was reduced when the content of CP in natural forage hay was below the lowest threshold level (7.0%) [34,35]. In the current study, the content of CP in the BG and CRG was higher than 7.0%, which implies that the contents of CP in these native forages can suffice the basic requirements of rumen microbial activity. The contents of NDF and ADF in forage are also considered the major factors affecting feed intake and feed conversion efficiency of ruminants, influencing animal performance [36,37]. In the current study, the content of NDF in the CRG and NO was lower than of the BG, and the content of ADF in the CRG was lower than in the BG and NO. This implies that CRG might have a higher rumen degradability than BG and NO [38].

Dry matter intake (DMI) is essential for dairy cows’ production performance. A critical factor affecting the DMI of dairy cows is the ruminal DM degradability, which is positively correlated with DMI [39]. The rumen DM degradation in roughage was affected by the content of cellulose and the degree of lignifications in the forage, which reflects the difficulty of degradation of the forage in ruminants [40]. This study showed a significant difference in the DM degradation between three kinds of forages. As the degradation time in the rumen prolongs, the DM degradation gradually increases and eventually stabilizes. Degradation trend of DM of three forages in the rumen was consistent with the results of Mehrez et al. [41]. The ED values of DM in the CRG and NO were higher than in the BG in our study than those reported by Moore-Colyer et al. [42] and Liu et al. [43]. These authors had reported that ED value of DM in the CRG and NO were 47% and 44.4%, respectively. The composition of NDF and ADF in roughage would affect the degradation rate of NDF and ADF in the rumen. There was differences between the content of NDF and ADF in the three forages, and this might be one of the factors that cause the NDF and ADF degradation in three forages [44]. The ED value of NDF and ADF in CRG was the highest, which might be due to the lower content of NDF and ADF and the higher content of CP in CRG [45]. In the present study, the ED value of NDF in CRG was 36.36%, which was close to the results documented by Xu et al. [46]. The ED value of ADF in CRG was 31.63%, which was slightly higher than in the study by Sun et al. [3] and the difference with current results of our study might be due to the types of experimental animals. The ADF is mainly composed of lignin, cellulose, and silicon dioxide and is thus considered the most indigestible part of roughage [47]. Due to the unique structure of lignin, it might have been difficult to be decomposed by rumen microorganisms [48]. Therefore, the ADF degradation in roughage was generally low [49]. The CP content of roughage in the rumen was mainly affected by the retention time and the difficulty of fermentation. In addition, it was also related to the characteristics of the roughage itself, such as the composition of CP, non-protein nitrogen (NPN) and the content of true protein, and the physical and chemical properties of true protein [50]. In the current study, the CP degradation of the CRG at different incubation time in the rumen was higher than of the BG and NO. The ED value of CP in the CRG documented in our study was slightly higher than the ED value reported by Bao et al. [51]. These slight variations in findings with Bao et al. [51] may be ascribed to the different experimental animals. The proportion of the potentially degradable fraction in the forage and its degradation rate could significantly affect the nutritional value [52]. In our findings, the rapidly degradable fraction of CP in the CRG was greater than in the BG, and the potentially degradable fraction of CP was greater than in the NO, so the ED value was higher than in the BG and NO.

Feed value was used to evaluate an important economic characteristic of roughage. The RFV (relative feed value) is a comprehensive reflection of NDF and ADF, and it was used to measure the quality of roughage. The RFV value and the nutritional value of the roughage were positively correlated. The RFV value of general high-quality roughage is assumed to be higher than 100 [25]. In the current study, the RFVs of the three forages were lower than 100, indicating that these three forages were low-quality roughage. RFQ was closer to the actual situation than RFV and could accurately classify forage. In this study, the RFV and RFQ of the CRG and NO were higher than of the BG. It is well known that TDN reflects the degradation characteristics of the roughage itself. In this study, the TDN and DDM values of the CRG were higher than of the BG and NO. The comprehensive analysis of our current study showed that CRG had the highest feeding value. Thus, it is recommended that CRG should be preferred as a roughage resource in ruminants feeding practice.

## 5. Conclusions

Altogether we concluded that there were differences in the nutrient contents of the three forages. The CRG had the highest CP and the lowest NDF and ADF contents. At the same time, the CRG had the highest effective degradation rate of DM, NDF, ADF, and CP in the rumen, and the values of TDN and DDM were higher than those in the BG and NO. The RFV and RFQ of the CRG and NO straw were higher than of the BG. Based on the present study, the CRG had the highest ruminal degradability and is, therefore, a promising alternative to utilize for feeding high yielding dairy cows.

## Figures and Tables

**Table 1:** Three forages (CRG, BG, and NO) information selected for the current study.

Items	Sample Description	Breed	Producing
CRG	Initial bloom stage	Dongbei	Zhaodong City, Heilongjiang Province
BG	Milk stage	Hordeum violaceum Boiss. Et Huet.	Gansu province
NO	Milk stage	Baiyan 3	Ulanqab City, Inner Mongolia Autonomous Region

**Table 2:** Composition and nutrient value of basic diet (DM basis %).

Items	Content	Nutrient Levels ^2^	Content
Ingredients		NE_L_ (MJ/Kg)	5.68
Chinese wildrye	6.38	CP	15.01
Alfalfa hay	20.31	EE	3.4
Oat hay	5.58	NDF	41.03
Wheat	1.76	ADF	26.69
Corn silage	24.48	Ca	0.55
Corn	3.66	P	0.39
Steam-flaked corn	13.52		
Soybean meal	6.55		
Extruded soybean	3.76		
Soybean hull	3.00		
Cottonseed	3.41		
Molasses	3.80		
Rumen-pass fatty acid	1.20		
Yeast powder	0.20		
Mycotoxin re-movement agent	0.06		
NaCl	0.31		
Limestone	0.32		
Ca(HCO_3_)_2_	0.34		
NaHPO_3_	0.67		
KHCO_3_	0.26		
Premix ^1^	0.31		
MgO	0.12		

^1^ Each kilogram of premix contains vitamin A 1,000,000 IU/kg, vitamin D 3,280,000 IU/kg, vitamin E 10,000 IU/kg, 1000 mg/kg nicotinic acid, 0.6 mg/kg copper, 1.2 mg/kg zinc, 2.2 mg/kg manganese, 76 mg/kg iodine, 5.5 mg/kg selenium, 29 mg/kg cobalt. ^2^ NE_L_ is a calculated value, while the other nutrient levels were measured values.

**Table 3:** Chemical composition of CRG, BG and NO (% DM basis).

Items	DM	CP	NDF	ADF	Ash	Starch
CRG	90.72	9.00	64.97	37.03	8.04	5.64
BG	90.95	8.90	67.33	37.93	8.53	2.05
NO	90.66	5.94	63.83	38.28	7.99	3.81

CRG: Chinese rye grass, BG: Barley grass, NO: Naked Oat, DM: dry matter (The dry matter content is calculated based on air-drying the sample), CP: crude protein, NDF: neutral detergent fiber, ADF: acid detergent fiber. The CP, Ash, NDF, ADF, and starch content are all calculated based on absolute dry matter.

**Table 4:** Rumen real-time degradability and degradation parameters of DM for three forages (CRG, BG, and NO).

Items	Real-Time Degradability Rate (%)								Degradation Parameter				
	4 h	8 h	12 h	24 h	30 h	36 h	48 h	72 h	a (%)	b (%)	c (%/h)	a + b (%)	ED (%)
CRG	12.62 ^b^	16.70 ^b^	20.04 ^b^	32.34 ^b^	39.13 ^c^	45.08 ^b^	50.42 ^c^	60.81	24.93 ^b^	38.50 ^a^	4.00 ^a^	63.43 ^a^	46.53 ^a^
BG	31.80 ^a^	35.07 ^a^	37.87 ^a^	48.27 ^a^	52.38 ^a^	55.24 ^a^	57.85 ^a^	60.33	27.42 ^a^	34.37 ^b^	2.00 ^b^	61.79 ^b^	40.89 ^b^
NO	30.98 ^a^	35.39 ^a^	36.72 ^a^	46.69 ^a^	48.34 ^b^	53.56 ^a^	54.03 ^b^	58.63	25.84 ^b^	35.87 ^a^	4.00 ^a^	61.71 ^b^	44.90 ^a^
SEM	0.64	0.67	0.60	1.19	0.98	1.04	0.97	1.57	0.97	2.83	0.00	3.04	0.58
*p*-Value	<0.001	<0.001	<0.001	<0.001	<0.001	<0.001	0.002	0.604	<0.001	<0.001	0.005	0.005	<0.001

In the same column, values with different letter mean significant difference (*p* < 0.05). “a” (%): rapidly degradable fraction; “b” (%): the potentially degradable fraction; “a + b” (%): total degradable fraction; “c” (%/h): the constant rate of degradation of “b” (%/h); ED (%): effective degradability. CRG: Chinese rye grass, BG: Barley grass, NO: Naked Oat. SEM: standard error of means.

**Table 5:** Rumen real-time degradability and degradation parameters of NDF for three forages (CRG, BG and NO).

Items	Real-Time Degradability Rate (%)								Degradation Parameter				
	4 h	8 h	12 h	24 h	30 h	36 h	48 h	72 h	a (%)	b (%)	c (%/h)	a + b (%)	ED (%)
CRG	9.88 ^a^	16.61 ^a^	16.61 ^a^	16.61 ^a^	38.73	43.00 ^a^	47.15 ^a^	50.68 ^b^	0.78	57.64 ^b^	5.00 ^a^	58.42 ^b^	36.36 ^a^
BG	9.76 ^a^	12.87 ^c^	12.87 ^c^	12.87 ^b^	37.96	42.82 ^a^	49.51 ^a^	52.85 ^a^	0.16	61.14 ^a^	3.00 ^b^	61.30 ^a^	30.68 ^b^
NO	8.58 ^b^	14.87 ^b^	14.87 ^b^	14.87 ^b^	36.29	38.86 ^b^	42.99 ^b^	48.41 ^c^	1.25	49.92 ^c^	4.00 ^ab^	51.17 ^c^	29.36 ^c^
SEM	0.34	0.42	0.76	0.67	0.96	1.03	0.75	0.67	0.60	0.82	0.00	1.20	0.30
*p*-Value	0.043	<0.001	<0.001	<0.001	0.239	0.031	0.001	0.004	0.473	<0.001	0.010	<0.001	<0.001

In the same column, values with different letter mean significant difference (*p* < 0.05). “a” (%): rapidly degradable fraction; “b” (%): the potentially degradable fraction; “a + b” (%): total degradable fraction; “c” (%/h): the constant rate of degradation of “b” (%/h); ED (%): effective degradability. CRG: Chinese rye grass, BG: Barley grass, NO: Naked Oat. SEM: standard error of means.

**Table 6:** Rumen real-time degradability and degradation parameters of ADF for three forages (CRG, BG, and NO).

Items	Real-Time Degradability Rate (%)								Degradation Parameter				
	4 h	8 h	12 h	24 h	30 h	36 h	48 h	72 h	a (%)	b (%)	c (%/h)	a + b (%)	ED (%)
CRG	9.63 ^a^	14.51 ^a^	24.85 ^a^	35.66 ^a^	37.19 ^a^	42.39 ^a^	48.48 ^a^	50.53 ^a^	0.74	52.33 ^b^	4.00 ^a^	53.07 ^a^	31.63 ^a^
BG	8.34 ^b^	12.39 ^b^	16.75 ^b^	30.52 ^b^	36.72 ^a^	41.59 ^a^	48.88 ^a^	51.15 ^a^	0.89	51.32 ^a^	3.00 ^b^	52.2 ^b^	26.13 ^c^
NO	8.13 ^b^	12.95 ^ab^	18.73 ^b^	31.90 ^b^	34.75 ^b^	37.65 ^b^	40.08 ^b^	46.80 ^b^	0.70	48.66 ^c^	4.00 ^ab^	48.73 ^c^	27.80 ^b^
SEM	0.33	0.61	0.82	1.16	0.58	0.73	0.99	0.53	0.34	0.99	0.00	1.24	0.38
*p*-Value	0.020	0.086	<0.001	0.031	0.035	0.003	<0.001	0.001	0.206	<0.001	0.053	0.001	<0.001

In the same column, values with different letter mean significant difference (*p* < 0.05). “a” (%): rapidly degradable fraction; “b” (%): the potentially degradable fraction; “a + b” (%): total degradable fraction; “c” (%/h): the constant rate of degradation of “b” (%/h); ED (%): effective degradability. CRG: Chinese rye grass, BG: Barley grass, NO: Naked Oat. SEM: standard error of means.

**Table 7:** Rumen real-time degradability and degradation parameters of CP for three forages (CRG, BG, and NO).

Items	Real-Time Degradability Rate (%)								Degradation Parameter				
	4 h	8 h	12 h	24 h	30 h	36 h	48 h	72 h	a (%)	b (%)	c (%/h)	a + b (%)	ED (%)
CRG	39.08 ^a^	42.89 ^a^	47.31	54.69 ^a^	59.04 ^a^	61.16 ^a^	64.95 ^a^	70.70 ^a^	34.69 ^a^	41.04 ^a^	3.00 ^b^	75.73 ^a^	54.46 ^a^
BG	30.54 ^c^	34.88 ^b^	44.82	51.44 ^b^	54.77 ^b^	58.52 ^ab^	60.45 ^b^	62.56 ^b^	21.57 ^b^	41.93 ^a^	6.00 ^a^	63.50 ^b^	48.54 ^b^
NO	36.6 ^b^	41.21 ^a^	44.32	52.35 ^ab^	53.62 ^b^	56.25 ^b^	60.34 ^b^	63.09 ^b^	32.43 ^a^	33.15 ^b^	4.00 ^b^	65.58 ^b^	50.34 ^b^
SEM	0.70	1.11	1.35	0.96	1.21	1.32	1.31	0.94	0.83	1.30	0.00	1.37	0.58
*p*-Value	<0.001	0.002	0.293	0.098	0.027	0.076	0.056	<0.001	<0.001	0.002	0.005	<0.001	<0.001

In the same column, values with different letter mean significant difference (*p* < 0.05). “a” (%): rapidly degradable fraction; “b” (%): the potentially degradable fraction; “a + b” (%): total degradable fraction; “c”(%/h): the constant rate of degradation of “b” (%/h); ED (%): effective degradability. CRG: Chinese rye grass, BG: Barley grass, NO: Naked Oat. SEM: standard error of means.

**Table 8:** Estimated feeding value of three forages (CRG, BG, and NO).

Items	TND (%)	DMI (%)	DDM (%)	RFV (%)	RFQ (%)
CRG	54.56	1.85	60.06	85.99	81.92
BG	53.87	1.78	59.35	82.02	78.09
NO	53.61	1.88	59.08	86.11	81.95

CRG: Chinese rye grass, BG: Barley grass, NO: Naked Oat. TDN: Total digestible nutrients, DMI: Dry matter intake, DDM: Digestible dry matter, RFV: Relative feed value, RFQ: Relative forage quality.

## Data Availability

All the data are already provided in the main manuscript.
